# Robust Conductive Hydrogels with Ultrafast Self-Recovery and Nearly Zero Response Hysteresis for Epidermal Sensors

**DOI:** 10.3390/nano11071854

**Published:** 2021-07-19

**Authors:** Xiuru Xu, Chubin He, Feng Luo, Hao Wang, Zhengchun Peng

**Affiliations:** 1 Guangdong Provincial Key Laboratory of Micro/Nano Optomechatronic Engineering, College of Mechatronics and Control Engineering, Shenzhen University, Shenzhen 518060, China; xiuruxu@foxmail.com (X.X.); LLF@szu.edu.cn (F.L.); 2 Center for Stretchable Electronics and Nano Sensors, School of Physics and Optoelectronic Engineering, Shenzhen University, Shenzhen 518060, China; hcbfighting@163.com

**Keywords:** hydrogels, toughness, wearable sensors

## Abstract

Robust conductive hydrogels are in great demand for the practical applications of smart soft robots, epidermal electronics, and human–machine interactions. We successfully prepared nanoparticles enhanced polyacrylamide/hydroxypropyl guar gum/acryloyl-grafted chitosan quaternary ammonium salt/calcium ions/SiO_2_ nanoparticles (PHC/Ca^2+^/SiO_2_ NPs) conductive hydrogels. Owing to the stable chemical and physical hybrid crosslinking networks and reversible non-covalent interactions, the PHC/Ca^2+^/SiO_2_ NPs conductive hydrogel showed good conductivity (~3.39 S/m), excellent toughness (6.71 MJ/m^3^), high stretchability (2256%), fast self-recovery (80% within 10 s, and 100% within 30 s), and good fatigue resistance. The maximum gauge factor as high as 66.99 was obtained, with a wide detectable strain range (from 0.25% to 500% strain), the fast response (25.00 ms) and recovery time (86.12 ms), excellent negligible response hysteresis, and good response stability. The applications of monitoring the human’s body movements were demonstrated, such as wrist bending and pulse tracking.

## 1. Introduction

Conductive hydrogels have shown great potential in wearable bioelectronic devices, human–machine interactions, health monitoring, flexible electronic skins, and medical bandages [1,2,3,4,5], owing to their softness, wetness, stretchability, biocompatibility, and wide tunable conductivity. However, the demands of practical versatility applications, such as load-bearing biosensors, soft robots, and real-time flexible wearable devices, require conductive hydrogels with further critical properties, such as robust mechanical performance, low hysteresis, fast self-recovery time, and rapid response to external stimuli. Traditional hydrogels usually show low toughness and tensile strength, long time self-recovery, and are easy to break due to fewer crosslinking joints and weaker interactions between molecular chains [6,7,8]. Therefore, investigating conductive hydrogels with rapid self-recovery, high tensile strength and toughness, and excellent elongation is still highly desired. Recently, Li et al. [9] reported a double network hydrogel from polyacrylamide (PAAm) and gelatin as frameworks. It showed 1.66 MPa tensile strength, 849% tensile strain, up to 1.5 S/m conductivity, and about 70% within 1 min self-recovery rate, but went along a large hysteresis. Wei et al. [10] utilized a hydrogel network by building weak non-covalent bonds and strong covalently cross-linked semiflexible electrospun fibrous nets. It exhibited about maximum 0.38 MPa tensile strength, ~1560 J/m^2^ toughness. It took 10 s to recover to 74% under 100% stretching-relaxing testing cycles. However, it still showed a large hysteresis with a dramatic decrease in the tensile strength after the first two cycles (from 0.07 MPa down to 0.01 MPa).

The chitosan quaternary ammonium salt (CQAS) is a natural macromolecule with good biocompatibility and biodegradability. In this work, we successfully prepared the acryloyl-grafted chitosan quaternary ammonium salt (acryloyl-grafted CQAS) crosslinking agent by the reaction between glycidyl methacrylate and the hydroxyl groups on the CQAS molecules. Subsequently, acrylamide as a monomer, acryloyl-grafted CQAS as a crosslinking agent, hydroxypropyl guar gum (HPG), and calcium chloride (CaCl_2_) were introduced into the network. Moreover, silica nanoparticles (SiO_2_ NPs) were applied to further enhance the mechanical properties of the as-prepared hydrogels.

## 2. Materials and Methods

### 2.1. Preparation of Acryloyl-Grafted Chitosan Quaternary Ammonium Salt (Acryloyl-Grafted CQAS)

First, 2.00 g of chitosan quaternary ammonium salt (CQAS, Mw: 50,000–100,000, from Coretests, Beijing, China) and 40 mL of deionized water were weighed into a 250 mL beaker. They were heated in a water bath at 70 °C and stirred for 2 h until the CQAS was dissolved completely. Next, we added 0.54 g of 3 mol/L NaOH aqueous solution (from Aladdin, Shanghai, China), and continued to stir it for 30 min to form a uniform reaction precursor. Then, 6 mL of glycidyl methacrylate (from Aladdin, Shanghai, China) was added into the reaction precursor, and reacted for 7 h under continuous stirring. After cooling down to room temperature, glacial acetic acid (from Aladdin, Shanghai, China) was used to adjust the solution to pH = 7, and then the reaction was stopped. The resulting product was then washed with dichloromethane (from Aladdin, Shanghai, China) and filtered. The filtered product was dissolved in deionized water and dialyzed with a dialysis bag for 7 days. Finally, the dialyzed product was freeze-dried to obtain the acryloyl-grafted chitosan quaternary ammonium salt (acryloyl-grafted CQAS).

### 2.2. Fabrication of PHC/Ca^2+^/SiO_2_ NPs Conductive Hydrogels

We weighted 5.0 g DI water, 1.2 g acrylamide monomer (AAm, 99.0%, from Aladdin, Shanghai, China), 0.03 g hydroxypropyl guar gum (HPG, from Baikang Chem., Shanghai, China), and 0.03 g calcium chloride (CaCl_2_, from Aladdin, Shanghai, China) into a clean glass bottle. The mixture was stirred and dissolved for 3 h at 70 °C. Then, different masses (0.008 g, 0.03 g, and 0.05 g) of silica nanoparticles (SiO_2_ NPs) were added and stirred for another 1 h at 70 °C. Next, it was cooled down to room temperature. Subsequently, 0.03 g of as-prepared acryloyl-grafted CQAS as the crosslinking agent and 0.03 g of ammonium persulfate (APS, from Aladdin, Shanghai, China) as the initiator were added, and dissolved evenly. Afterward, the mixture was treated by ultrasound for 5 min to remove air bubbles. The solution was poured into the templates. Then, it was cured at 70 °C for 100 min and then cooled down to room temperature. Finally, the PAAm-HPG/acryloyl-grafted CQAS/Ca^2+^/SiO_2_ NPs hydrogels (PHC/Ca^2+^/SiO_2_ NPs) was well prepared.

### 2.3. Characterization and Methods

The chemical bonding structure of the as-prepared samples was analyzed by a Nicolet 6700 spectrometer (Thermo Fisher Scientific, Waltham, MA, USA) with a total reflection infrared spectroscopy (ATR-IR) accessory in the range of 400–4000 nm. The morphology and elemental analysis were characterized by scanning electron microscopy (SEM, Shimadzu SSX-550, Tokyo, Japan), attached with an energy dispersive X-ray spectrometer (EDX, Oxford, Abingdon, UK). The samples were cut to 17 mm (length) × 6 mm (width) × 0.3 mm (thickness). The strain sensing performance was obtained using a tensile machine (Instron E1000, Norwood, MA, USA), attached with a digital multimeter (34465A, Keysight Technologies, Santa-Rosa, CA, USA). We calculated the elastic modulus and toughness based on the slope of the linear part in the stress-strain curve and by integrating the area of the stress-strain curve, respectively. 

## 3. Results

### 3.1. Preparation and Characterization of PHC/Ca^2+^/SiO_2_ NPs Conductive Hydrogels

**Figure 1:**
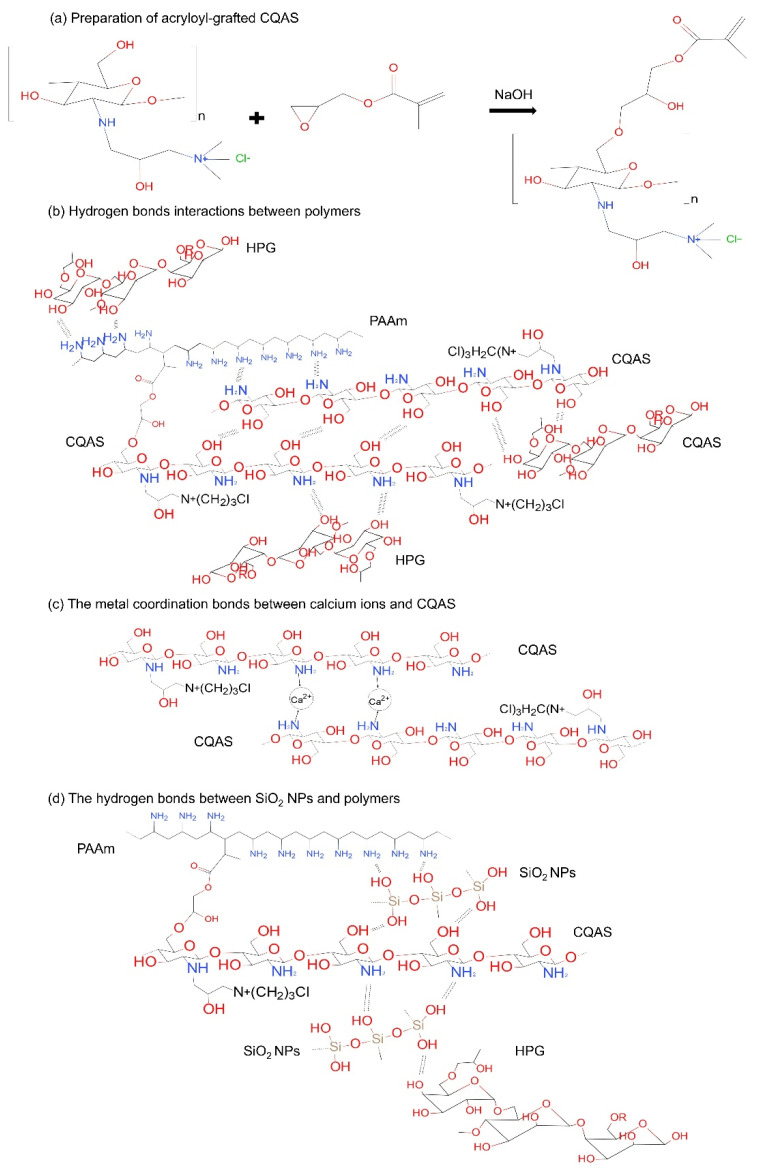
(**a**) The scheme of preparation of the acryloyl-grafted CQAS. Illustration of the internal crosslinked networks of the as-prepared PHC/Ca^2+^/SiO_2_ NPs hydrogels of (**b**) hydrogen bonds interactions between polymers, (**c**) metal coordination between calcium ions and acryloyl-grafted CQAS and (**d**) interactions of SiO_2_ NPs with PAAm and HPG polymers.

As an essential material for on-skin wearable electronics, the biocompatibility property of conductive hydrogels has gained more and more attention [11,12]. Low-toxic crosslinking agents and natural polymers are ideal choices for the preparation of hydrogels. Chitosan quaternary ammonium salt (CQAS) is a natural macromolecular polymer with good water solubility, antibacterial properties, film-forming properties, compatibility, and biodegradability. Since the surface of the CQAS is rich in hydroxyl groups, functional groups can be grafted to the surface of the CQAS molecules. Modification with functional groups of a crosslinking agent for hydrogels can effectively improve their polymerization. The hydroxyl groups on the CQAS molecules can undergo a ring-opening polymerization reaction with the epoxy group of glycidyl methacrylate under alkaline conditions [13]. The acryloyl groups can be grafted onto the surface of the CQAS to obtain an acryloyl-grafted macromolecular crosslinking agent (Figure 1a). Subsequently, we used PAAm and hydroxypropyl guar gum (HPG) as the hydrogel backbone, acryloyl-grafted CQAS as the crosslinking agent, and APS as the initiator to prepare a series of PAAm-HPG/acryloyl-grafted CQAS hydrogels (PHC) by thermally initiated free radical in-situ polymerization. Ca^2+^ was introduced into the gel, which not only provided conductive ions, but also can form metal ion bonds with acryloyl-grafted CQAS to increase the strength and toughness of the hydrogel. To further improve the mechanical property, SiO_2_ NPs were applied to the conductive hydrogels for reinforcement. A large number of hydrogen bond interactions were formed between polymers (PAAm, HPG, and acryloyl-grafted CQAS) (Figure 1b), metal coordination between Ca^2+^ and acryloyl-grafted CQAS (Figure 1c), and SiO_2_ NPs nano-reinforcement (Figure 1d) in the as-prepared PHC/Ca^2+^/SiO_2_ NPs conductive hydrogels, resulting a strong and reversible physical-chemical cross-linked networks in the hydrogel. Therefore, the PHC/Ca^2+^/SiO_2_ NPs conductive hydrogels are expected to have excellent mechanical properties.

**Figure 2:**
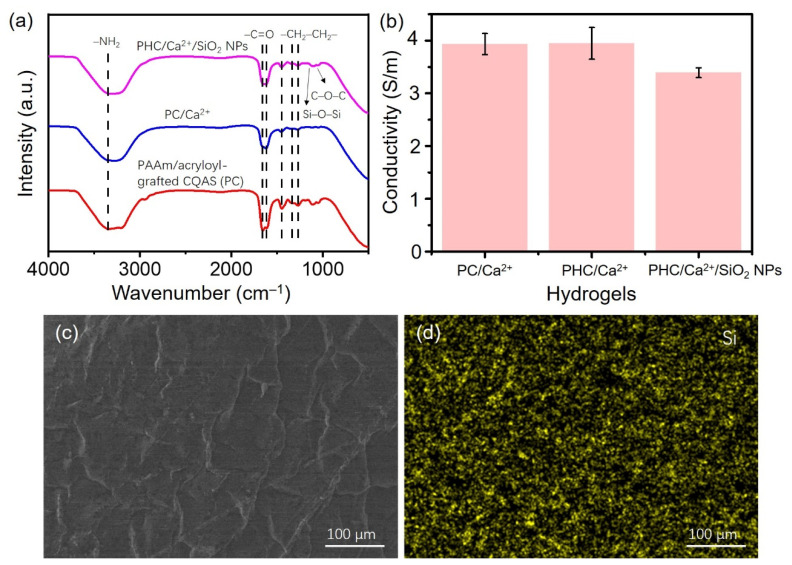
(**a**) FT-IR spectra and (**b**) conductivity of different conductive hydrogel samples. (**c**) The SEM image and (**d**) the element mapping (Si) of EDX spectra of a freeze-dried PHC/Ca^2+^/SiO_2_ NPs hydrogels.

The FT-IR spectra (Figure 2a) has been investigated, and it showed that –C=O in the amide groups was found at 1607 cm^−1^. Peaked around 3355 cm^−1^, 1450 cm^−1^, 1656 cm^−1^ were ascribed to the stretching vibration of –NH_2_, –CH_2_–CH_2_–, –C=O in the amide groups. Bands at 1349 cm^−1^ were assigned to the C–H stretching vibration of -CH_3_ in the quaternary ammonium salt. The peak at 1264 cm^−1^ was ascribed to the stretching vibration of β-1,4 glycosidic bonds of CQAS, which indicated that the chemically cross-linked PAAm/acryloyl-grafted CQAS network was successfully formed. The C–O–C stretching vibration peak of the glucose ring on the HPG was found at 1049 cm^−1^, and the stretching vibration peak of Si–O–Si was found at 1114 cm^−1^ [14,15,16], which showed that PHC/Ca^2+^/SiO_2_ NPs conductive hydrogel was prepared successfully by the thermally initiated free radical in situ polymerization. The presence of CaCl_2_ benefited the as-prepared PHC/Ca^2+^/SiO_2_ NPs hydrogels with good ionic conductivity without adding additional conductive agents. We can see that both PAAm/acryloyl-grafted CQAS/Ca^2+^ (denoted as PC/Ca^2+^) and PAAm-HPG/acryloyl-grafted CQAS/Ca^2+^ (denoted as PHC/Ca^2+^) conductive hydrogels showed good conductivity, and their conductivity can reach ~3.947 S/m in Figure 2b. There was only a slight decrease of PHC/Ca^2+^/SiO_2_ NPs samples with a conductivity of ~3.390 S/m. The existence of Si component, as well as a quite uniform distribution of SiO_2_ NPs in the PHC/Ca^2+^/SiO_2_ NPs hydrogels was indicated, from the SEM and corresponding EDX map of Si element in Figure 2c,d.

### 3.2. Mechanical Properties of PHC/Ca^2+^/SiO_2_ NPs Conductive Hydrogels

**Figure 3:**
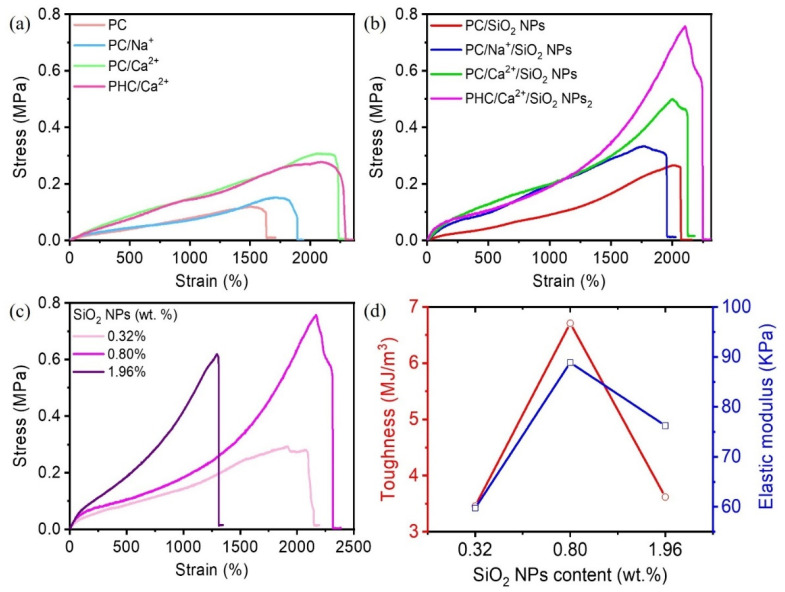
(**a**) The strain–stress curves of different conductive hydrogel samples (**a**) before and (**b**) after adding SiO_2_ NPs. (**c**) The strain–stress curves and (**d**) the corresponding elastic modulus and toughness of PHC/Ca^2+^/SiO_2_ NPs conductive hydrogels with different SiO_2_ nanoparticle contents.

The mechanical properties of different hydrogel samples are compared in Figure 3. It showed that the PAAm/acryloyl-grafted CQAS hydrogel (PC) could be stretched to 16 times its original length. However, its tensile strength was as low as 100 kPa (in Figure 3a). Subsequently, we incorporated different metal ions (Na^+^ and Ca^2+^) into the PAAm/acryloyl-grafted CQAS (PC) hydrogel. Both the addition of Na^+^ and Ca^2+^ effectively improved the elasticity and tensile strength of the resulted PC/Na^+^ and PC/Ca^2+^ hydrogels. The fracture strain and tensile strength of PC/Ca^2+^ hydrogels were 1.2 and 2 times larger than that of PC/Na^+^ hydrogels, respectively, indicating stronger metal coordination between Ca^2+^ and acryloyl-grafted CQAS than that between Na^+^ ions and acryloyl-grafted CQAS. Additionally, the polymer HPG further improved the elasticity of the PC/Ca^2+^ conductive hydrogel, which can be stretched to about 2,294% strain (PHC/Ca^2+^). Moreover, it was found in Figure 3b that the fracture strain and tensile strength of each hydrogel sample was significantly improved due to the incorporation of SiO_2_ NPs. The tensile strength of the PHC/Ca^2+^/SiO_2_ NPs hydrogel was the highest, 2.74 times larger than that of the PHC/Ca^2+^ samples, which proved strengthening and toughening effects the PHC/Ca^2+^/SiO_2_ NPs conductive hydrogels by SiO_2_ NPs.

We further evaluated the influence of different SiO_2_ NPs contents on the mechanical properties of the PHC/Ca^2+^/SiO_2_ NPs conductive hydrogels (Table 1). Figure 3c showed that as the weight ratio of SiO_2_ NPs increased, the tensile strength of the PHC/Ca^2+^/SiO_2_ NPs hydrogels increased from 0.301 MPa (0.32 wt.% of SiO_2_ NPs) to 0.765 MPa (0.80 wt.% of SiO_2_ NPs), and then decreased to 0.619 MPa (1.96 wt.% of SiO_2_ NPs). Similarly, the fracture strain of the resultant PHC/Ca^2+^/SiO_2_ NPs conductive hydrogels also increased from 2149% to 2256%, and then decreased to 1292%. We calculated the corresponding toughness and elastic modulus from the strain–stress curve (Figure 3c) in Figure 3d. The toughness and elastic modulus of the PHC/Ca^2+^/SiO_2_ NPs conductive hydrogel increased first and then decreased with the increasing content of SiO_2_ NPs. It was found that when the content of SiO_2_ NPs was 0.80 wt.%, the PHC/Ca^2+^/SiO_2_ NPs hydrogel showed the best fracture strain (2256%), tensile strength (0.765 MPa), toughness (6.71 MJ/m^3^), and elastic modulus (88.86 kPa).

The excellent mechanical performance of the as-prepared PHC/Ca^2+^/SiO_2_ NPs conductive hydrogel can be explained for the following reasons. Firstly, acryloyl-grafted CQAS as a macromolecular crosslinking agent can help to realize the effective chemical crosslinking of the PHC/Ca^2+^/SiO_2_ conductive hydrogels, which can increase the stretchability and elasticity of the conductive hydrogel networks. Secondly, Ca^2+^ can form strong metal coordination interactions with acryloyl-grafted CQAS and can spontaneously form metal-ligand chelates, further improving the strength of the PHC/Ca^2+^/SiO_2_ NPs conductive hydrogel. Thereby, an excellent physical crosslinking network was formed with strong hydrogen bond interactions between the polymers (PAAm, HPG, and CQAS), the metal ion interactions between acryloyl-grafted CQAS and Ca^2+^, and the SiO_2_ nano-reinforcement. Those non-covalent bonds effectively improved the energy dissipation of the conductive hydrogel under large strains, so that the PHC/Ca^2+^/SiO_2_ NPs conductive hydrogel showed better toughness [17,18].

**Figure 4:**
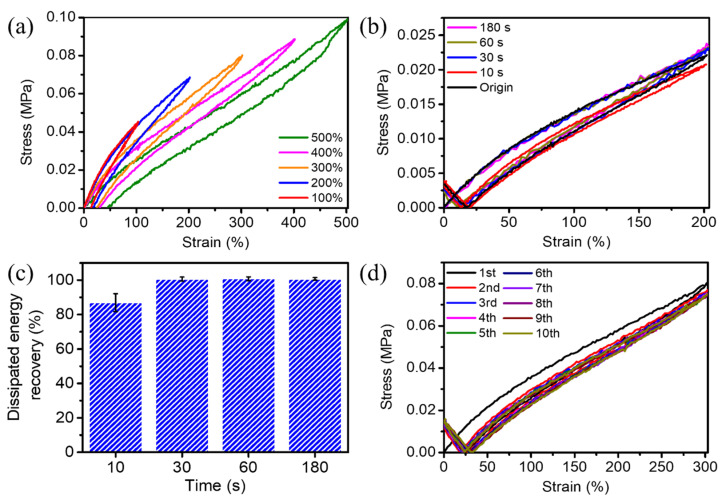
(**a**) The cyclic loading–unloading mechanical performance of PHC/Ca^2+^/SiO_2_ NPs conductive hydrogels under different strains. (**b**) Cyclic stress–strain test at a strain of 200% with different rest intervals, and (**c**) the corresponding dissipated energy recovery ratio. (**d**) The ten times continuous loading-unloading tensile test at 300% applied strain with no relaxation time between each cycle.

The cyclic loading–unloading mechanical performance of the PHC/Ca^2+^/SiO_2_ NPs hydrogel is shown in Figure 4. The loading-unloading curves exhibited a 13% hysteresis of the PHC/Ca^2+^/SiO_2_ NPs hydrogel under 100% strain (Figure 4a). When it was stretched to a more significant strain, the tensile stress gradually increased, indicating a good energy dispersion. When the hydrogel was stretched, it changed the conformation of the polymer molecular chains. The formed non-covalent bonds in the hydrogel broke gradually, to provide the required energy for the friction between the polymer segments. When an external force was applied, it could help to overcome the internal friction between the chain segments as well, and the stretched molecular chains were curled up again and restored to their original states. Normally, it requires quite a long time for conventional hydrogels to overcome the friction caused by the external forces to achieve movements. In this work, the PHC/Ca^2+^/SiO_2_ NPs conductive hydrogel showed a relatively rapid self-recovery performance (100% recovery to its original state within 30 s), which indicated that the as-prepared hydrogels could dissipate energy effectively. Furthermore, we performed cyclic loading-unloading experiments of the PHC/Ca^2+^/SiO_2_ NPs conductive hydrogels under 200% strain at 25 °C with different relaxation time intervals (10 s, 30 s, 60 s, 180 s), as shown in Figure 4b. As the relaxation time interval increased, the hysteresis area of the hydrogel gradually went to its original state. Figure 4c showed that the PHC/Ca^2+^/SiO_2_ NPs hydrogel could recover 80% of its dissipated energy after standing for 10 s, and 100% that of within 30 s. It indicated that those broken reversible bonds could reform quickly within a short relaxation time and exhibited good self-recovery properties. To further evaluate the anti-fatigue performance of PHC/Ca^2+^/SiO_2_ NPs hydrogel, we investigated ten cycles continuous stretching-relaxation tensile tests from 0% to 300% applied strain, with no relaxation time between each cycle. As shown in Figure 4d, the maximum tensile stress of the hydrogel reached 0.08 MPa in the first cyclic tensile test. As the stretching cycles went on, the maximum tensile stress of the hydrogel continued to decrease and finally reached a plateau. The maximum stress of the hydrogel could still maintain 93.5% of the initial maximum stress after ten cycles of tensile loading-unloading with a strain of 300%. The self-recovery and other properties of reported typical conductive hydrogels are summarized and compared with our major results in Table 2. Our PHC/Ca^2+^/SiO_2_ NPs hydrogel showed outstanding self-recovery, conductivity, and maximum gauge factor performance.

### 3.3. Electro-Mechanical Performance of PHC/Ca^2+^/SiO_2_ NPs Conductive Hydrogels

**Figure 5:**
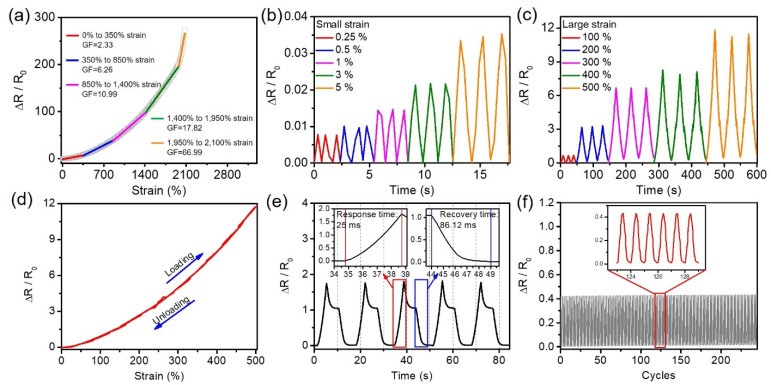
(**a**) The classic strain response curve of the PHC/Ca^2+^/SiO_2_ NPs conductive hydrogel and the corresponding linear fitting results. The cyclic loading-unloading test of PHC/Ca^2+^/SiO_2_ NPs conductive hydrogel under different (**b**) small strains of 0.25%, 0.5%, 1%, 3%, and 5% and (**c**) large strains of 100%, 200%, 300%, 400%, and 500%, respectively. (**d**) The ΔR/R_0_ curves of PHC/Ca^2+^/SiO_2_ NPs conductive hydrogel under the applied strain of 500% at a stretching rate of 100 mm/min. (**e**) Response time and recovery time of the PHC/Ca^2+^/SiO_2_ NPs hydrogel under tensile strain of 200%. (**f**) PHC/Ca^2+^/SiO_2_ NPs conductive hydrogels underwent a 200% strain loading-unloading cycles at room temperature.

To verify the strain sensing performance of the PHC/Ca^2+^/SiO_2_ NPs conductive hydrogels, we tested the relative resistance change as a function of the strains (where R was the resistance after stretching and R_0_ was the initial resistance before stretching). It was found from Figure 5a that the relative resistance changes in the PHC/Ca^2+^/SiO_2_ NPs conductive hydrogel increased as the strain increased. According to the linear fitting results (Figure 5a), the strain response curve of PHC/Ca^2+^/SiO_2_ NPs conductive hydrogel can be divided into five regions, including: 0–350%, 350–850%, 850–1400%, 1400–1950%, 1950–2100%. The calculated gauge factors (GF) of those five regions were 2.33, 6.26, 10.99, 17.82, and 66.99, respectively. Those results were better than most existing PAAm hydrogel-based strain sensors [27,28,29]. The relative resistance change of the PHC/Ca^2+^/SiO_2_ NPs conductive hydrogel under different strains can be found in Figure 5b,c. The hydrogel showed the ability to monitor small strains (0.25% to 5%) and large strains (100% to 500%). The conductive hydrogel exhibited stable and repetitive signals under different strains. The minimum detectable strain of the PHC/Ca^2+^/SiO_2_ NPs conductive hydrogel was 0.25% strain, showing a low strain-sensing sensitive detection limit. Benefiting from the reversible elastic networks, the hydrogel achieved a negligible response hysteresis from 0% to 500% strain (Figure 5d), which is one of the desired critical properties of strain sensors. Figure 5e showed a rapid strain response with response and recovery time of 25.00 ms and 86.12 ms, respectively. Additionally, the relative resistance changes of the hydrogel showed stability performance during 300 cycles (Figure 5f).

**Figure 6:**
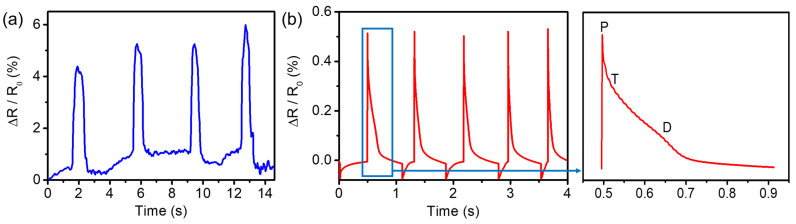
(**a**) Sensing performance of PHC/Ca^2+^/SiO_2_ NPs conductive hydrogels in response to (**a**) wrist bending and (**b**) to pulse sensing properties and the amplified waveform of pulse.

The as-prepared PHC/Ca^2+^/SiO_2_ NPs conductive hydrogel was applied as an epidermal sensor for flexible and wearable testing. It was demonstrated to monitor both large-scale and tiny human motions. We attached the PHC/Ca^2+^/SiO_2_ NPs conductive hydrogel to the wrist with tape to detect wrist bending movements on the relative resistance change of the hydrogel. As shown in Figure 6a, when we bent the wrist downward, the conductive hydrogel underwent stretching, and the rate of relative resistance changes of the hydrogel increased. When the equilibrium position of the hydrogel was restored, the rate of relative resistance changes of the hydrogel decreased. We tested four cycles and found that the performance was relatively stable. Figure 6b showed that the PHC/Ca^2+^/SiO_2_ NPs conductive hydrogel was attached to the wrist to track heart pulse rates. As shown in Figure 6b, each signal peak represented a heart beating process. About five signal peaks were displayed, and the pulse beat showed a beating rate of about 75 times/min, which met the regular pulse rate of adults. The three peaks of P, T, and D were found in Figure 6b, which was also in line with the waveform diagram of the radial artery pulse signal at the wrist in the previous related reports. Based on the above results, we believe that PHC/Ca^2+^/SiO_2_ NPs conductive hydrogels can realize real-time pulse tracking and provide support for clinical therapeutic decisions.

## 4. Conclusions

In summary, high performance strain sensors based on the nanoparticles enhanced PHC/Ca^2+^/SiO_2_ NPs conductive hydrogels were demonstrated. Owing to their stable chemical and physical hybrid crosslinking networks and the formation of a large number of reversible non-covalent interactions, the presented sensors exceed the performance of previously reported conductive hydrogels in terms of ultrafast self-recovery capability, high gauge factor, negligible response hysteresis, good elasticity and conductivity. The formation and broken of non-covalent interactions during the stretching-relaxation process was discussed. We believe it will present a new paradigm for personalized medical monitoring, intelligent electronic skins, and in vivo nerve electrodes in the future.

## Figures and Tables

**Table 1:** The mechanical properties of PHC/Ca^2+^/SiO_2_ NPs conductive hydrogels containing different SiO_2_ NPs contents.

PHC/Ca^2+^/SiO_2_ NPs Conductive Hydrogels	Fracture Strain (%)	Tensile Strength (MPa)	Toughness (MJ/m^3^)	Elastic Modulus (kPa)
0.00 wt.% SiO_2_ NPs	2,294	0.278	3.21	19.99
0.32 wt.% SiO_2_ NPs	2,149	0.301	3.46	59.79
0.80 wt.% SiO_2_ NPs	2,256	0.765	6.71	88.86
1.96 wt.% SiO_2_ NPs	1,292	0.619	3.62	76.23

**Table 2:** Brief summary of results reported on conductive hydrogels.

Hydrogels	Conductivity (S/m)	Self-Recovery Rate	Maximum Gauge Factor
Nanoclay/NAGA/GelMA [19]	-	12 h (100%)	-
CMC/Fe^3+^/PAAm/SMA/NaCl/SDBS [20]	1.82	2 h (100%)	4.02
chitosan/PAAm/Na_2_SO_4_ or chitosan/PAAm/Na_3_Cit [21]	-	4 h (>90%)	-
AMP/Q-chitosan/NaCl/PAAm [22]	2.8	1 h (95.4%)	3.38
Agar/PAAm/stearyl methacrylate/SDS/NaCl [23]	2	2 min (99.6%)	-
positively charged imidazolium-based IL monomers with urea groups/SMAP/KCl [24]	3	2 h (100%)	-
PAAm/PAA-Fe^3+^/NaCl [25]	0.72	4 min (100%)	1.96
PAAm/Gelatin/Na_3_Cit [9]	1.5	1 min (70%)	2.04
CMCS/Ca^2+^/PAAm/PNMA [26]	2.688	5 min (83%)	9.18
This work	3.39	30 s (100%)	66.99

NAGA: N-acryloyl glycinamide; GelMA: gelatin methacryloyl; CMC: carboxymethyl cellulose; PAAm: polyacrylamide; SMA: stearyl methacrylate; NaCl: sodium chloride; SDBS: sodium dodecyl benzene sulfonate; CS: chitosan; Na_3_Cit: sodium citrate; AMP: adenosine monophosphate; Q-chitosan: quaternized chitosan; PAA: polyacrylic acid; SMAP: 3-sulfopropyl methacrylate potassium salt; KCl: potassium chloride; Na_2_SO_4_: sodium sulfate; IL: ionic liquid. CMCS: carboxymethyl chitosan; PNMA: poly(N-methylol acrylamide).
